# Is the Effect of Body Dissatisfaction on Depressive Symptoms Dependent on Weight Status? A Study with Early-to-Middle Adolescents

**DOI:** 10.3390/ejihpe10040072

**Published:** 2020-11-03

**Authors:** Maria João Carapeto, Raquel Domingos, Guida Veiga

**Affiliations:** 1 Departamento de Psicologia, Escola de Ciências Sociais, Universidade de Évora, 7005-345 Évora, Portugal; 2 Departamento de Desporto e Saúde, Escola de Ciências e Tecnologia, Universidade de Évora, 7005-345 Évora, Portugal; m44623@alunos.uevora.pt (R.D.); gveiga@uevora.pt (G.V.); 3 Comprehensive Health Research Centre (CHRC), University of Évora, 7005-345 Évora, Portugal

**Keywords:** body image, body mass index, overweight, internalizing, body image self-discrepancy

## Abstract

Depression is a recognized mental health problem in adolescence and body dissatisfaction is an important risk factor. The main goal of this study is to examine the relationship between body dissatisfaction and depressive symptoms, and whether it depends on adolescents’ weight status, an issue that remains understudied. Two hundred and fourteen adolescents (12–16 years) completed self-report measures of depressive symptoms, body dissatisfaction and weight status (i.e., current body weight and height, to compute body mass index z-scores, BMIz). Hierarchical multiple regression and moderation analyses were conducted, accounting for gender and age effects on depressive symptoms. Body dissatisfaction was found to be a predictor of depressive symptoms for the low and median BMIz adolescents, but not for those with high BMIz. In addition, this interaction of body dissatisfaction and BMIz improved the ability of the regression model to explain depressive symptoms´ variance beyond the effect of gender and age. The high-BMIz adolescents presented higher body dissatisfaction but similar levels of depressive symptoms, compared to the lower BMIz adolescents. These findings suggest the influence of body dissatisfaction in the emergence of depressive symptoms in the first half of adolescence, and the importance of weight status throughout this path.

## 1. Introduction

The risk of depression sharply increases as children enter adolescence, especially for girls [1,2]. Depression is a recognized serious public health concern, associated with downstream negative consequences in the short term (e.g., academic difficulties, suicidal ideation and risk behaviors) [3,4,5] and in the long term (e.g., lower income levels, higher divorce rates, suicidality, depression and other mental health problems in adulthood) [6,7,8]. The existing knowledge about its consequences and the epidemiological data showing an alarming increase in the risk of depression in the recent generations of adolescents [9,10] require the understanding of the risk factors for adolescents’ depression. This understanding is of paramount importance when planning policies and designing prevention and treatment plans. To date, several risk factors for adolescents’ depression have been proposed, such as insecure attachment patterns [11]; emotion dysregulation [12]; cognitive biases and other cognitive vulnerabilities [13,14]; co-rumination [15]; particular aspects in self-concept organization [16]; stressful events [14,17]; biological factors [18]; among others. 

Body image has also been pointed out as a critical risk factor for adolescents’ depression [19]. Body image refers to a “multifaceted psychological experience of embodiment, especially but not exclusively one’s physical appearance. (…) It encompasses one’s body-related self-perceptions and self-attitudes, including thoughts, beliefs, feelings, and behaviors” [20] (p. 1–2). Body (dis)satisfaction is a facet of body image [20] referring to a cognitive and affective (dis)appreciation of own body, globally considered or focused on specific body features (weight, shape, particular body parts, etc.) [21]. Thus, body dissatisfaction refers to negative appreciations about one’s own body. This specific dimension of body image has been pointed as a risk factor for several health-related problems, such as quality of life [22], risky health behaviors (smoking, drug use, self-harm, etc.) [23], eating disorders [24], and depression [24,25,26,27,28]. 

In a period of life marked by significant bodily changes [29], adolescents internalize the sociocultural standards of body image. These standards are transmitted to the young people in contemporary societies by the parents and peers, mass media and social networks, and value specific body ideals, often challenging to attain [30]; girls desire thinner bodies [31,32], and boys desire larger muscles [31,33]. Body (dis)satisfaction is related to one’s perception of own body meeting these socially prescribed body ideals [30] and can be conceptualized regarding the discrepancy between the self-perceived actual and ideal body [21,34]. Throughout adolescence, the perception of the real and the ideal body changes, and body dissatisfaction increases [32,33,35]. 

The self-perceived actual body does not always provide an accurate picture of the body weight status [34]. Adolescent girls tend to overestimate their bodies’ size, and boys tend to underestimate it [36]. Research on the relationship between body dissatisfaction and weight status consistently shows that overweight adolescents, especially obese, report more body dissatisfaction [37,38,39,40]. However, this relationship seems to be dependent on gender; for instance, underweight girls report the highest levels of body satisfaction [38], whereas underweight boys show body dissatisfaction [39].

The conceptualization of body dissatisfaction as the self-discrepancy between the perceived actual and ideal body brings some light to understanding the link between body dissatisfaction and depressive symptoms. According to Higgins’ self-discrepancy theory [41], the person describes him/herself as she/he is in reality (i.e., actual self) and according to self-guides, such as the ideal self (i.e., how he/she would like to be). Greater actual–ideal self-discrepancies promote a sense that a positive outcome has not been accomplished and dejection affects, dysphoria, or sadness. Simultaneously, high actual–ideal self-discrepancies have self-regulatory functions and motivate the activation of mechanisms to reduce such discrepancies [41]. In general, the link between actual–ideal self-discrepancies and depressive symptoms has received extensive support [16,42,43], including the specific self-discrepancy about the body [44] (for a review, see [34]), especially when dissatisfaction is due to an actual body larger than the ideal [44]. In other words, according to the self-discrepancy theory [41], when people fail to meet their ideals, a negative affect emerges, thus favoring the emergence and maintenance of depressive symptoms. Actual–ideal self-discrepancies also promote the motivation for a set of behaviors that can bring the body closer to the ideal (e.g., diets, physical exercise, etc.) [34]. 

Although the relationship between body dissatisfaction and depressive symptoms is a consistent finding, there is a lack of studies examining the role of weight status in this relationship. Contrary to body dissatisfaction, weight status has not been consistently related to depressive symptoms. Some studies reported that overweight and obese individuals report higher levels of depression [45,46] and that underweight girls present high levels of depression [45], whereas others showed no differences in depression levels for different weight status [19,40]. 

To date, only a few studies have focused on weight status (mostly body mass index, BMI) as a moderator of the effect of body dissatisfaction on adolescents’ depressive symptoms. A study with a large sample of Swiss adults found that increased body dissatisfaction is related to increased depressive symptoms, independent of BMI [47]. However, a study involving Chinese adolescents showed that weight status had a moderator effect on the association between body dissatisfaction and depressive symptoms, such that the stronger association was verified for the underweight adolescents [48]. Besides, a more recent study [40] revealed that body dissatisfaction positively predicted depressive symptoms among overweight/obese adolescents and vice-versa; however, among the healthy weight group, there was no relation in either direction [40]. 

Several studies claim that the alarming increase in depression, body dissatisfaction, and overweight/obesity in adolescents is a serious public health problem [23,49]. Despite the research showing body dissatisfaction as a predictor of depression in adolescence, studies on the role of weight status in this relationship are still scarce and inconsistent. Hence, this cross-sectional study aims to examine the effect of body dissatisfaction on depressive symptoms and the moderator role of weight status, beyond the effects of gender and age. A second aim is to examine the effect of gender, age and the interaction between age and gender on depressive symptoms.

## 2. Materials and Methods

### 2.1. Participants

The sample of this study included a total of 214 adolescents (92 boys, 122 girls), aged between 12 and 16 years (Mean = 13.50, SD = 1.121). Adolescents were attending four different schools from the South of Portugal. Eighty-one (37.9%) participants were in the 7th grade, 65 in the 8th grade (30.4%), and 68 (31.8%) in the 9th grade. The predominant levels of mothers’ and fathers’ education were higher education (46.7% and 35.5%, respectively) and secondary education (30.8% and 26.6%, respectively). 

### 2.2. Instruments and Measures

A sociodemographic questionnaire was administered to characterize participants regarding gender, age, grade, parental education, body weight, and height, among others. 

#### 2.2.1. Depressive Symptoms

A measure of depressive symptoms was obtained through the Depression subscale of the Portuguese version [50] of the Depression, Anxiety and Stress Scale (DASS) [51]. This 7-item self-report subscale measures depressive complaints felt in the previous week (e.g., *I couldn’t seem to experience any positive feeling at all*). Items are rated on a 4-point scale from 0 (*did not apply to me at all*) to 3 (*applied to me very much, or most of the time*). A total score was obtained by computing the sum of all 7 items, ranging from 0 to 21. Higher results reveal more negative affective states. The internal consistency of the subscale was good (Cronbach’s α = 0.85). 

#### 2.2.2. Body Dissatisfaction

Body dissatisfaction was assessed using the Portuguese version [52] of the Contour Drawing Rating Scale (CDRS) [53], a figural drawing scale consisting of two sequences of silhouettes, one for males and other for females, ordered from 1 (the thinnest) to 9 (the largest). The individual is asked to identify the figure that most identifies with his/her current appearance and the figure that corresponds to his/her ideal appearance. Body dissatisfaction was computed as the absolute value of the difference between the scores of the actual and ideal body appearances (ranging from 0 to 8). A second, categorical variable, named “body dissatisfaction direction”, was also computed in order to capture the direction of the body image dissatisfaction. The raw difference between actual and ideal appearances ranges from −8 (maximum body dissatisfaction, ideal appearance is larger than the actual) to +8 (maximum body dissatisfaction, ideal appearance is thinner than the actual), where 0 corresponds to satisfaction with body appearance. Five categories were created: satisfied with body image (value 0), very dissatisfied due to larger body ideals (values −2 and lower), dissatisfied due to larger body ideals (value −1), dissatisfied due to thinner body ideals (value +1), very dissatisfied due to thinner body ideals (values +2 and higher). For the entire sample (N = 214), actual body image was associated with BMI (r = 0.70, *p* < 0.01) and body dissatisfaction was associated with BMI (r = 0.45, *p* < 0.01).

#### 2.2.3. Weight Status

Weight status was obtained through body mass index (BMI) that is measured by dividing the body weight by the square of the body height (thus, expressed in units of kg/m^2^). Adolescents were asked to report their weight and height. Previous studies have reported the good reliability of self-reported weight and height (e.g., [27,54,55]), which have also been argued as a valid methodological approach in the context of epidemiological studies [56]. Besides, students are aware of their own weight and height, as they often register these measures in Physical Education classes. Body mass index z-scores (BMIz) were computed as a measure of relative weight-for-height adjusted for age and gender, following the World Health Organization (WHO) international reference data [57], and using the WHO AnthroPlus software [58].

### 2.3. Procedures

This study was part of a larger study that was conducted in accordance with the Declaration of Helsinki and approved by the Ethics Committee for Scientific Research in the Areas of Human Health and Well-Being of the University of Évora, Portugal (Document No. 18033). The consent to collect the data in the schools was obtained from the Portuguese Ministry of Education and from the schools’ principals, who gave permission to start the study. Written consent from the adolescents and the responsible adults for their education (mostly parents) was obtained. With the teachers’ permission, questionnaires were handed out to the adolescents during class time by one of the authors of this study, taking about 25 minutes to be filled out. Two hundred and twenty-four adolescents completed the questionnaires. Ten individuals were excluded from the sample because they did not provide data about weight, height, or perceived actual or ideal appearance in the CDRS.

### 2.4. Statistical Analyses

All statistical analyses were performed with IBM SPSS (version 24.0). As missing data regarding depression accounted for only 1.9%, imputation using the median by nearby points values technique was performed [59]. 

Descriptive statistics (frequencies, means, SD) were calculated for depression, body dissatisfaction (quantitative variable), BMIz, gender and age. In order to test the association between variables, *Pearson* correlations were computed as well as t-tests for gender differences.

In order to examine the relationship between body dissatisfaction and depressive symptoms and, specially, whether that relationship depends on the level of BMIz, a hierarchical regression analysis was conducted, with depressive symptoms as dependent variable in three nested models. In Model 1, gender, age and gender × age interaction were the predictors. Model 2 added body dissatisfaction and BMIz, and Model 3 added the body dissatisfaction × BMIz interaction. Whenever the interaction terms were significant (*p* < 0.05), the moderation analysis was completed with the Hayes’ Process macro v. 3.5 for SPSS [60] to probe and visualize the interaction effects on depressive symptoms. For the gender × age interaction, gender was entered as predictor and age as a moderator, controlling for body dissatisfaction, BMIz and their interaction term. For the body dissatisfaction × BMIz interaction, body dissatisfaction was entered as a predictor and BMIz as a moderator, controlling for gender, age and gender × age. The conditional effects of body dissatisfaction on depressive symptoms were tested at three values of the moderators (age and BMIz, respectively), which were set to the percentiles 16th, 50th and 84th and represent low, median and high values, respectively. Two graphs were prepared to support the interpretation of the moderation effects of age and BMIz, respectively, depicting the slopes at the low, median and high levels of the moderator. In addition, the Johnson-Neyman technique was used to a more precise identification of the region(s) of statistical significance, i.e., the values of the moderators (age or BMIz) for which the predictor (gender or body dissatisfaction) had a significant conditional effect on depressive symptoms. Age, body dissatisfaction and BMIz were mean-centered in all regression analysis and gender was coded as dummy (males 0, females 1). Assumptions for regression analysis were checked [61]. 

Finally, a categorical variable of weight status was created using the moderation analyses’ conditioning values of BMIz as cut-points to define three categories: low BMIz, values equal or lower than the BMIz value at the 16th percentile; median BMIz, values between 16th and 84th percentiles; and high BMIz, values equal or higher that 84th percentile. Frequencies were computed and chi-squares were carried out to test the association between BMIz categories and gender and with the categorical variable of body dissatisfaction direction. Besides, analysis of variance was performed to test differences in body dissatisfaction, depressive symptoms and age between the three weight status categories, and Bonferroni multiple comparisons were used to identify the particular weight status categories involved in the previously identified differences. For all statistical analyses significance was set at *p* < 0.05.

## 3. Results

### 3.1. Descriptive Statistics and Associations among Variables

As Table 1 shows, no gender differences were found in age, BMIz, body dissatisfaction and depressive symptoms. Yet, a marginally significant (*p* = 0.077) difference emerged for BMIz such that boys presented slightly higher values than girls. Only body dissatisfaction (*p* = 0.036) was associated with age, showing that as adolescents grow older body dissatisfaction increase.

Body dissatisfaction showed to be positively and significantly associated with BMIz (*p* = 0.000) and depressive symptoms (*p* = 0.007). Depressive symptoms level was not significantly associated with BMIz.

### 3.2. Predicting Depressive Symptoms: Hierarchical Multiple Regression and Moderation Analysis

Table 2 shows the change statistics of the three nested models in the hierarchical regression performed, the statistics of the predictors of depressive symptoms under analysis and the adjustment statistics for each model. 

Model 1 was statistically significant (*p* = 0.049), explaining 2% of the depressive symptoms variance, due to the interaction of gender and age (*p* = 0.007), since neither gender nor age separately were statistically significant predictors. When body-related variables (i.e., body dissatisfaction and BMIz) were added to the model, its explanatory power increased (*p* = 0.046), as suggested by the change statistics in Table 2. Yet, Model 2 explained 4% of the variability of depressive symptoms (*p* = 0.015), and body dissatisfaction contributed positively, but only marginaly, to the prediction of depressive symptoms (*p* = 0.059), while BMIz did not. Finally, the inclusion of the interaction between body dissatisfaction and BMIz in Model 3 significantly improved (*p* = 0.030) the explanatory power of the previous model explaining 6% of the variance of depressive symptoms (*p* = 0.005) (a small to medium effect size) [62]. Along with the predictors previously found (interaction between gender and age, and, marginally, body dissatisfaction), the interaction between body dissatisfaction and BMIz was also shown to be a significant predictor.

**Figure 1:**
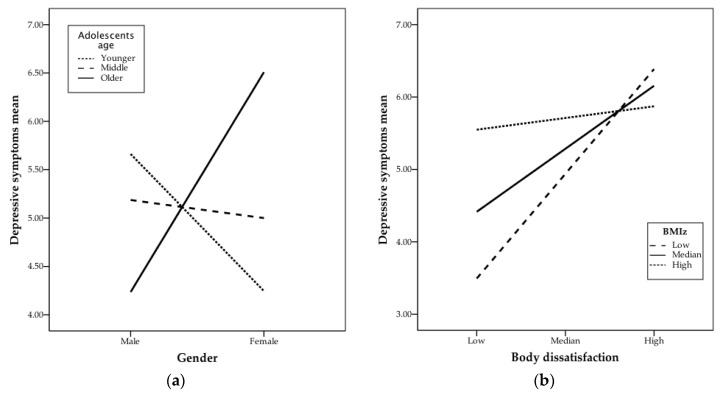
Simple slopes of moderation analysis: (**a**) relationship between gender and depressive symptoms at three age levels, younger (16th percentile, 12 years), median (50th percentile, 13 years) and older (84th percentile, 15 years) adolescents, statistically significant for older adolescents, *p* = 0.037; (**b**) relationship between body dissatisfaction and depressive symptoms at three levels of BMIz (body mass index z-scores), low (16th percentile, BMIz = −0.86), median (50th percentile, BMIz = 0.13) and high (84th percentile, BMIz = 1.35), statistically significant for low, *p* = 0.004, and median, *p* = 0.012, BMIz. Values of body dissatisfaction were Low = 0, Median = 1.25 and High = 2.

Following the significant Gender × Age effect, the conditional effects of gender on depressive symptoms for the three age levels are presented in Table 3 and illustrated in Figure 1a. The conditional effect of gender on depressive symptoms is negative but statistically non-significant for the younger adolescents (16th percentile: mean-centered value is −1.50, and row value for age is 12.00), as well as for adolescents with a median age (50th percentile, age mean-centered is −0.50, and row value for age is 13.00). For the older adolescents (86th percentile, age mean-centered value is 1.50, and row value for age is 15.00), gender revealed a positive conditional effect on depressive symptoms, such that older girls had higher levels of depressive symptoms than boys. More precisely, the Johnson-Neyman technique indicated that the relationship between gender and depressive symptoms was only significant when age was higher than 1.12 (value mean-centered), or 14.62 years old.

The interaction effects between body dissatisfaction and BMIz are depicted in Figure 1b. Table 3 presents the conditional effects of body dissatisfaction on depressive symptoms for low, median and high BMIz. For low (16th percentile, mean-centered value = −1.01, BMIz = −0.86) and median (percentile 50th, mean-centered value = −0.02, BMIz = 0.13) levels of BMIz, the effect of body dissatisfaction on depressive symptoms was positively significant. However, for high levels of BMIz (percentile 84th; mean-centered value = 1.20, row value = 1.35), the effect of body dissatisfaction on depressive symptoms was not significant. In a more detailed perspective, the Johnson-Neyman technique indicates that the relationship between body dissatisfaction and depressive symptoms was only significant when BMIz was lower than 0.54 (or 0.39, value mean-centered), which applies to 64.49% of the sample (138 participants). 

Table 4 shows that the adolescents of the three weight status (BMIz) categories defined by the values of BMIz at the 16th and 84th percentiles identified in the moderation analysis as cut-off values between categories (Low: BMIz < −0.86, N = 34; Median: BMIz from −0.86 to 1.35, N = 146; High: BMIz > 1.35, N = 34) are similar in terms of age and levels of depressive symptoms but differ in terms of their body dissatisfaction. The Bonferroni method for pairwise multiple comparisons revealed that high-BMIz adolescents were significantly more dissatisfied with their bodies than the low (M difference = 1.44, se = 0.24, *p* = 0.000), and median BMIz adolescents, M difference = 1.16, se = 0.19, *p* = 0.000. Concerning gender, no significant associations with weight status categories were found, χ^2^ (214, 2) = 2.83, *p* = 0.243, φ = 0.115. 

Figure 1b illustrates the moderating effect of BMIz on the relationship between body dissatisfaction and depressive symptoms. The three different slopes represent the relationship between body dissatisfaction and depressive symptoms for the three BMIz levels. The slopes of the median and the low BMIz show that, for these BMIz levels, an increase in body dissatisfaction predicts an increase in depressive symptoms. However, the less pronounced slope regarding the high BMIz level illustrates the non-significant relationship between body dissatisfaction and depressive symptoms.

Table 5 shows the frequency of adolescents in each weight status category, by category of body dissatisfaction direction. Weight status and the direction of body dissatisfaction categories were significantly associated, χ^2^ (214, 8) = 87.79, *p* = 0.000, φ = 0.64. Fifty percent of the low BMIz adolescents were dissatisfied with their bodies and would like to be larger, while 47% were satisfied with their bodies. Only 3% were dissatisfied and desired to be thinner. Among the adolescents with a median BMIz, the majority (53%) were dissatisfied because they would like to be thinner, and 16% were dissatisfied because they would like to be larger. Only 32% were satisfied with their median size body. Regarding the high-BMIz adolescents, none was satisfied with their body, and, with one exception, everyone was dissatisfied and desired to be thinner.

## 4. Discussion

The aim of this study was to examine the extent to which body dissatisfaction and BMIz are related to adolescents’ depressive symptoms, as well as gender and age. Indeed, body dissatisfaction, especially its interaction with BMIz, along with the interaction between age and gender, was found as a predictor of adolescents’ depressive symptoms. In addition, these body-related variables seemed to have an effect on depressive symptoms that goes beyond the effect of the gender and age interaction.

The results suggest that neither gender nor age alone influenced adolescents’ depression levels. Instead, the effect of gender on depressive symptoms was dependent on adolescents’ age. Specifically, gender differences in the severity of depressive symptoms only appeared for older adolescents (above 14.62 years), with girls presenting higher levels than boys. Although other studies have also shown no gender [19,50] and age-related [19,50,63] differences, most studies have revealed an increase in depressive symptoms throughout adolescence and gender differences starting in the earlier years of adolescence [1,64,65]. Possibly, the short age range of our sample may have limited our ability to capture an onward increase in depressive symptoms over adolescence and the gradual emergence of gender differences, until these became more expressive in the older adolescents of the sample. Accordingly, other studies found that this gender gap only appears at 14 years of age [66] or that this difference increases from middle to late adolescence [67]. Besides, the lack of gender differences of the younger groups might also be explained by the characteristics of the instrument used to measure depression. First, DASS’s depression scale was originally designed to measure adults’ symptoms; thus, it might not be sensitive to the particular experience of depression over adolescence [68]. Second, its reduced number of items might impair the sensitivity to the patterns of depressive symptoms distinctively experienced by adolescent boys and by adolescent girls [69,70].

The present findings go along with previous studies suggesting that body dissatisfaction is a predictor of depressive symptoms [25,26,27,28]. This relationship can be explained by the self-discrepancy theory [41], as body dissatisfaction was measured as the discrepancy between the actual and the ideal body perceived by the adolescents. Hence, such actual–ideal body self-discrepancy might signal the absence of positive outcomes as the attainment of the ideal body, therefore promoting dejection affects that can originate and maintain depressive symptoms [41,42]. Besides, higher actual–ideal self-discrepancies could motivate behaviors to approach the ideal body, such as dieting and other weight control strategies [34]. The increase in body dissatisfaction with age found in this study is also consistent with other research. Bucchianeri and colleagues [35] attributed this association to the increase in body size (BMI) in adolescence. However, we did not find an association between BMIz and age, possibly because we used the standardized BMI z-scores [57] adjusted for gender and age differences in the weight-for-height. 

In line with other studies, BMIz was not a significant predictor of depressive symptoms [19]. However, BMIz was found to moderate the relationship between body dissatisfaction and depressive symptoms [40,48]. That is, the effect of body dissatisfaction on depressive symptoms was dependent on weight status, such that body dissatisfaction only predicted depressive symptoms of adolescents with low and median BMIz (more precisely, when BMIz < 0.54). However, our study, along with the few other studies that have addressed the moderating role of BMI in adolescents’ depressive symptoms, does not show consistent findings. Wang and colleagues [40], who examined black American adolescents, only found a positive association between body dissatisfaction and depressive symptoms for overweight/obese adolescents. In contrast, Chen and colleagues [48], who examined Chinese adolescents, showed that body dissatisfaction was more strongly related to depressive symptoms among underweight adolescents and that this relationship was non-significant for overweight males. As a whole, our findings, along with those from Wang and colleagues [40] and Chen and colleagues [48], suggest a possible cultural influence on the relationship between body dissatisfaction, weight status, and depressive symptoms. Possibly, weight status has a different impact on adolescents’ self-appreciation of their bodies across different cultures. This lack of consistency in findings may also be related to the different research methods used. For example, while the present study followed a cross-sectional design and measured body dissatisfaction as a discrepancy between the actual and ideal body indicated on a figural scale, others investigated the relationship between dissatisfaction and depressive symptoms prospectively [40], and measured body dissatisfaction through verbal self-report questionnaires [48].

In line with previous research [40], adolescents with high BMIz were more dissatisfied with their own bodies and more likely to desire to be thinner (only one adolescent did not, and none was satisfied with his/her body) than their counterparts. However, contrary to other studies [46], adolescents with high BMIz did not present significantly higher levels of depressive symptoms, and body dissatisfaction lost its predictive power on depression levels of adolescents with a high BMIz. Possibly, these adolescents believed that the ideal body would be attainable within a reasonable time and therefore their actual–ideal body self-discrepancies motivated behaviors to reduce weight [34,41] (even unhealthy behaviors; [71]); in this scenario, the increment of negative affect could be buffered, at least temporarily (see, for instance, [72]). On the other hand, a long lasting lack of success in attaining the ideal body might give place to an adaptive decrease in the importance of body appearance within the self-concept, which refrains a further increase in negative affect [19,73]. This could also contribute to understanding the "fat and jolly" hypothesis on obese adolescents presented elsewhere [74]. However, more research is needed to replicate the present findings and clarify why the higher levels of body dissatisfaction of high-BMIz adolescents did not predict higher depression levels. 

The fact that body dissatisfaction lost its predictive power on depression levels of adolescents with a high BMIz further suggests that other factors, except body dissatisfaction, might play an important role in these adolescents’ depression levels. For example, peer victimization [75] or inflammation [76] have been shown to have a critical role in high BMI adolescents’ depression levels, which denotes the need for further research. Furthermore, there are other factors that can contribute to depressive symptoms in this group of adolescents, such as reduced physical activity, sedentary behavior or poor diet [77].

Overall, the full model was able to explain a modest 6% of the variance in depressive symptoms; therefore, space was left to the role of other explaining factors of adolescents’ depressive symptoms, as referred in the paragraph above, in the Introduction Section and elsewhere [78].

### Limitation of this Study and Implications for Practice and Future Research

This study has some limitations. First, the sample size did not allow for a more detailed moderation analysis, impeding the disentangling of the effects of age and gender on the mediation role of BMIz. Second, despite the good psychometric properties and the extensive use in adolescents’ research, possibly the used instruments could not fully account for some developmental specificities of both body image and depressive symptoms, specifically in what concerns gender. Regarding the body dissatisfaction assessment, adolescents might find it difficult to identify themselves (and their ideal bodies) with the generalized and schematic CDRS silhouettes [79]. This concern is especially relevant for boys, as the silhouettes hardly reflect the muscularity usually desired [31,33]. Third, the cross-sectional design of our study clearly recommends caution in the conclusions regarding developmental changes and causality. Moreover, although previous studies have shown that using self-reported anthropometric measurements is a reliable method to calculate BMI for weight classification purposes [27,54,55,56,80], one should admit that, for some adolescents, self-reporting BMI might be biased. 

In this context, the present results are promising and encourage further research with larger samples and a wider age range, to allow the simultaneous analysis of other moderators’ effects on the relationship between body dissatisfaction and depressive symptoms throughout adolescence (e.g., gender, age) (see, for instance, supplementary analyses with this sample in Appendix A, Table A1 and Table A2). Longitudinal and prospective studies are also welcome. Careful attention is needed for the selection of instruments, sensitive to the different patterns of depressive symptomatology in developing adolescent boys and girls [69,70], and to the particular body concerns of adolescents boys and girls [31,81]. Besides, research is needed to clarify the interplay between body dissatisfaction, weight status and other possible variables in their contribution to adolescents’ depressive symptoms, especially in overweight and obese adolescents. Finally, despite being beyond the scope of this study, there are two important topics that deserve further research. One is the relationship between BMI and body dissatisfaction that some authors have suggested to follow no-linear patterns that are different by gender [82]. The other is the relationship between BMI and depression [45]. 

As implications for practice in clinical and educational contexts with adolescents, this study reinforces the importance of including body dissatisfaction in preventing and treating depression [83]. In addition, this study cautions to the possible inadequacy or, at least, the limitations of the interventions focused on body dissatisfaction to prevent or treat depression, when intended to overweight and obese adolescents. 

## 5. Conclusions

This study has brought some new light to the less studied issue of the influence of body dissatisfaction in the emergence and maintenance of depressive symptoms in the first half of adolescence, particularly concerning the moderating role of weight status, and the moderation role of age in gender differences in depressive symptoms. The relationship between gender and depressive symptoms was found to be dependent on age (i.e., gender differences appeared only for the older adolescents, 14.6 to 16 years), and the effect of body dissatisfaction on depressive symptoms was found to be moderated by weight status level (i.e., body dissatisfaction influenced depression level but only for low to median BMIz, not for adolescents with high BMIz). These results encourage further research and have the potential to inspire improvements in preventive and treatment interventions addressing adolescents’ depression. 

## Figures and Tables

**Table 1:** Descriptive statistics, *t* test statistics for gender differences and correlations (Pearson).

Variables	Min–Max	Mean (SD)		*t* (212)	*p*	Cohen’s *d*	1	2	3
		Boys	Girls						
1–Age	12–16	13.62 (1.19)	13.40 (1.06)	1.41	0.160	0.20	-		
2–BMIz	−3.94–2.67	0.31 (1.10)	0.03 (1.13)	1.78	0.077	0.25	−0.00	-	
3–Body dissatisfaction	0–8	1.10 (1.15)	1.15 (1.07)	−0.33	0.744	0.05	0.14 *	0.38 **	-
4–Depressive symptoms	0–21	4.97 (4.96)	5.25 (4.73)	−0.42	0.677	0.06	0.04	0.13	0.19 **

*Note.* Boys, N = 92; Girls, N = 122. Min–Minimum; Max–Maximum; BMI–body mass index; BMIz–body mass index z-score. ** *p* < 0.01. * *p* < 0.05.

**Table 2:** Hierarchical multiple regression for predicting depressive symptoms (N = 214).

Models and Predictors	Δ*F* (*df*1, *df*2)	Δ*R*^2^	*B*	*SE*	*t*	*p* ^a^	Adjusted *R*^2^	*F* *(df1, df2)*	*p*
**Model 1**	2.66 (3210)	0.037				0.049	0.02	2.66 (3210)	0.049
Gender ^b^			0.30	0.66	0.45	0.653			
Age			−0.63	0.42	−1.51	0.132			
Gender × Age			1.59	0.59	2.72	0.007			
**Model 2**	3.14 (2208)	0.028				0.046	0.04	2.88 (5208)	0.015
Gender			0.32	0.66	0.48	0.632			
Age			−0.62	0.42	−1.48	0.141			
Gender × Age			1.39	0.59	2.38	0.018			
Body dissatisfaction			0.62	0.32	1.90	0.059			
BMIz			0.24	0.32	0.77	0.442			
**Model 3**	4.76 (1207)	0.021				0.030	0.06	3.24 (6207)	0.005
Gender			0.43	0.66	0.65	0.516			
Age			−0.48	0.42	−1.14	0.257			
Gender × Age			1.23	0.59	2.10	0.037			
Body dissatisfaction			0.86	0.34	2.52	0.012			
BMIz			0.28	0.32	0.87	0.383			
Body dissatisfaction × BMIz			−0.58	0.27	−2.18	0.030			

^a^ The *p* value in the “Model 1”, “Model 2” and “Model 3” lines refers to change statistics.

^b^ Males = 0, Females = 1.

**Table 3:** Moderation analysis for predicting depressive symptoms: conditional effect of the predictors at specific values of the moderator (N = 214).

Moderator /Levels	*B*	*SE*	*t*	*p*
Age of adolescents ^a^				
Younger (16th percentile, 12 years)	−1.42	1.11	−1.27	0.204
Middle (50th percentile, 13 years)	−0.19	0.73	−0.26	0.798
Older (84th percentile, 15 years)	2.27	1.08	2.10	0.037
BMIz ^b^				
Low (16th percentile, −0.86)	1.45	0.50	2.90	0.004
Median (50th percentile, 0.13)	0.87	0.34	2.54	0.012
High (84th percentile, 1.35)	0.16	0.38	0.42	0.674

^a^ Moderates the effect of the gender (predictor) on depressive symptoms, controlling for body dissatisfaction, BMIz and their interaction term.

^b^ Moderates the effect of body dissatisfaction (predictor) on depressive symptoms, controlling for gender, age and gender × age.

**Table 4:** Means (SD) for depression, body dissatisfaction and age, by weight status (BMIz) category.

	BMIz categories			F (2, 211)	*p*	η^2^*_p_*
	Low	Median	High			
Age	13.06 (1.01)	13.47 (1.12)	14.06 (1.04)	0.80	0.452	0.007
Body dissatisfaction	0.71 (0.76)	0.99 (0.95)	2.15 (1.37)	21.91	0.000	0.172
Depression	4.65 (5.09)	5.02 (4.71)	6.06 (5.03)	0.84	0.434	0.008

*Note.* Low: BMIz < −0.86, N = 34; Median: BMIz from −0.86 to 1.35, N = 146; High: BMIz > 1.35, N = 34.

**Table 5:** Count (% within weight status category) of the cases in the categories of weight status (BMIz), by category of body dissatisfaction direction.

BMIz Categories	Direction of body dissatisfaction categories n (%)						
	Satisfied	Very Dissatisfied Ideal Large	Dissatisfied Ideal Large	Total Dissatisfied Ideal Large ^a^	Dissatisfied Ideal Thin	Very Dissatisfied Ideal Thin	Total Dissatisfied Ideal Thin ^b^
Low BMIz	16 (47.1)	6 (17.6)	11 (32.4)	17 (50)	1 (2.9)	0 (0)	1 (2.9)
Median BMIz	46 (31.5)	3 (2.1)	20 (13.7)	23 (15.8)	51 (34.9)	26 (17.8)	77 (52.7)
High BMIz	0 (0)	0 (0)	1 (2.9)	1 (2.9)	11 (32.4)	22 (64.7)	33 (97.1)
Total	62 (29.0)	9 (4.2)	32 (15.0)	41 (19.2)	63 (29.4)	48 (22.4)	111 (51.9)

*Note.* Low: BMIz < −0.86, N = 34; Median: BMIz, from −0.86 to 1.35, N = 146; High: BMIz > 1.35, N = 34.

^a^ Sums the frequencies of previous columns “Very dissatisfied, Ideal large” and “Dissatisfied, Ideal large”.

^b^ Sums the frequencies of previous columns “Dissatisfied, Ideal thin” and “Very dissatisfied, Ideal thin”.

## Appendix A

Moderation analyses were performed with the Hayes’ Process macro v. 3.5 for SPSS [60] separately by gender. Depressive symptoms entered as a dependent variable, body dissatisfaction as a predictor and BMIz as a moderator. Body dissatisfaction and BMIz were mean-centered.

**Table A1:** Multiple regression for predicting depressive symptoms.

Predictors	Females (N = 122)				Males (N = 92)			
	*B*	*SE*	*t*	*p*	*B*	*SE*	*t*	*p*
Body dissatisfaction	1.15	0.44	2.58	0.011	0.94	0.55	1.72	0.090
BMIz	0.38	0.41	0.92	0.361	−0.02	0.173	−0.11	0.916
Body dissatisfaction × BMIz	−0.27	0.37	−0.72	0.471	−0.25	0.12	−2.06	0.042

*Note.* BMIz – body mass index z-scores.

Model Summary for males (Table A1), *R*^2^ = 0.06, *F* (3, 88) = 1.73, *p* = 0.167, and change provided by the interaction term, Δ*R*^2^ = 0.05, *F* (1, 88) = 4.25, *p* = 0.042.

Model Summary for females (Table A1), *R*^2^ = 0.09, *F* (3, 118) = 4.09, *p* = 0.008, and change provided by the interaction term, Δ*R*^2^ = 0.00, *F* (1, 118) = 0.52, *p* = 0.471.

**Table A2:** Conditional effects of body dissatisfaction at specific values of BMIz, for predicting depression in males.

BMIz Levels ^a^	*B*	*SE*	*T*	*p*
Low (16th percentile, −2.46)	1.62	0.76	2.13	0.036
Median (50th percentile, −0.29)	1.08	0.58	1.86	0.067
High (84th percentile, 3.87)	0.05	0.51	0.11	0.914

^a^ Values for Low, Median and High BMIz are raw scores.

The Johnson-Neyman technique indicates that, for males, the relationship between body dissatisfaction and depressive symptoms was only significant when BMIz was lower than −1.03 (or −1.34, value mean-centered; 36.97% of the males sample).

## References

[1] Cohen J.R., Andrews A.R., Davis M.M., Rudolph K.D. (2018). Anxiety and depression during childhood and adolescence: Testing theoretical models of continuity and discontinuity. J. Abnorm. Child Psychol..

[2] Merikangas K.R., He J.-P., Burstein M., Swanson S.A., Avenevoli S., Cui L., Benjet C., Georgiades K., Swendsen J. (2010). Lifetime prevalence of mental disorders in U.S. adolescents: Results from the National Comorbidity Survey Replication--Adolescent Supplement (NCS-A). J. Am. Acad. Child Adolesc. Psychiatry.

[3] Riglin L., Petrides K.V., Frederickson N., Rice F. (2014). The relationship between emotional problems and subsequent school attainment: A meta-analysis. J. Adolesc..

[4] Zhang W., Zhang L., Chen L., Ji L., Deater-Deckard K. (2019). Developmental changes in longitudinal associations between academic achievement and psychopathological symptoms from late childhood to middle adolescence. J. Child Psychol. Psychiatry.

[5] Fredrick S.S., Demaray M.K., Malecki C.K., Dorio N.B. (2018). Can social support buffer the association between depression and suicidal ideation in adolescent boys and girls?. Psychol. Sch..

[6] Auerbach R.P., Kim J.C., Chango J.M., Spiro W.J., Cha C., Gold J., Esterman M., Nock M.K. (2014). Adolescent nonsuicidal self-injury: Examining the role of child abuse, comorbidity, and disinhibition. Psychiatry Res..

[7] Auerbach R.P., Tsai B., Abela J.R.Z. (2010). Temporal relationships among depressive symptoms, risky behavior engagement, perceived control, and gender in a sample of adolescents. J. Res. Adolesc..

[8] Johnson D., Dupuis G., Piche J., Clayborne Z., Colman I. (2018). Adult mental health outcomes of adolescent depression: A systematic review. Depress. Anxiety.

[9] Bor W., Dean A.J., Najman J., Hayatbakhsh R. (2014). Are child and adolescent mental health problems increasing in the 21st century? A systematic review. Aust. N. Z. J. Psychiatry.

[10] Mojtabai R., Olfson M., Han B. (2016). National trends in the prevalence and treatment of depression in adolescents and young adults. Pediatrics.

[11] Brumariu L.E., Kerns K.A. (2010). Parent-child attachment and internalizing symptoms in childhood and adolescence: A review of empirical findings and future directions. Dev. Psychopathol..

[12] Gonçalves S.F., Chaplin T.M., Turpyn C.C., Niehaus C.E., Curby T.W., Sinha R., Ansell E.B. (2019). Difficulties in emotion regulation predict depressive symptom trajectory from early to middle adolescence. Child Psychiatry Hum. Dev..

[13] Smith E.M., Reynolds S., Orchard F., Whalley H.C., Chan S.W. (2018). Cognitive biases predict symptoms of depression, anxiety and wellbeing above and beyond neuroticism in adolescence. J. Affect. Disord..

[14] Hankin B.L., Abramson L.Y. (2001). Development of gender differences in depression: An elaborated cognitive vulnerability–transactional stress theory. Psychol. Bull..

[15] Stone L.B., Hankin B.L., Gibb B.E., Abela J.R.Z. (2011). Co-rumination predicts the onset of depressive disorders during adolescence. J. Abnorm. Psychol..

[16] Carapeto M.J., Feixas G. (2019). Self-knowledge and depressive symptoms in late adolescence: A study using the repertory grid technique. J. Constr. Psychol..

[17] Griffith J.M., Long E.E., Young J.F., Hankin B.L. (2020). Co-occurring trajectories of depression and social anxiety in childhood and adolescence: Interactive effects of positive emotionality and domains of chronic interpersonal stress. Abnorm. Child Psychol..

[18] Sequeira M.E., Lewis S.J., Bonilla C., Smith G.D., Joinson C. (2017). Association of timing of menarche with depressive symptoms and depression in adolescence: Mendelian randomisation study. Br. J. Psychiatry.

[19] Murray K., Rieger E., Byrne D. (2018). Body image predictors of depressive symptoms in adolescence. J. Adolesc..

[20] Cash T.F. (2004). Body image: Past, present, and future. Body Image.

[21] Menzel J.E., Krawczyk R., Thompson J.K., Cash T.F., Smolak L. (2011). Attitudinal assessment of body image for adolescents and adults. Body Image. A Handbook of Science, Practice, and Prevention.

[22] Griffiths S., Murray S.B., Bentley C., Gratwick-Sarll K., Harrison C., Mond J.M. (2017). Sex differences in quality of life impairment associated with body dissatisfaction in adolescents. J. Adolesc. Health.

[23] Bornioli A., Lewis-Smith H., Smith A., Slater A., Bray I. (2019). Adolescent body dissatisfaction and disordered eating: Predictors of later risky health behaviours. Soc. Sci. Med..

[24] American Psychiatric Association (2013). Diagnostic and Statistical Manual of Mental Disorders, DSM-5.

[25] Morken I.S., Røysamb E., Nilsen W., Karevold E.B. (2019). Body dissatisfaction and depressive symptoms on the threshold to adolescence: Examining gender differences in depressive symptoms and the impact of social support. J. Early Adolesc..

[26] Paxton S.J., Neumark-Sztainer D., Hannan P.J., Eisenberg M.E. (2006). Body dissatisfaction prospectively predicts depressive mood and low self-esteem in adolescent girls and boys. J. Clin. Child Adolesc. Psychol..

[27] Sharpe H., Patalay P., Choo T.H., Wall M., Mason S.M., Goldschmidt A.B., Neumark-Sztainer D. (2018). Bidirectional associations between body dissatisfaction and depressive symptoms from adolescence through early adulthood. Dev. Psychopathol..

[28] Flores-Cornejo F., Kamego-Tome M., Zapata-Pachas M.A., Alvarado G.F. (2017). Association between body image dissatisfaction and depressive symptoms in adolescents. Braz. J. Psychiatry.

[29] Simmons R.G., Blyth D.A. (2017). Moving into Adolescence: The Impact of Pubertal Change and School Context.

[30] Tiggemann M., Cash T.F., Smolak L. (2011). Sociocultural perspectives on human appearance body image. Body Image. A Handbook of Science, Practice, and Prevention.

[31] Karazsia B.T., Murnen S.K., Tylka T.L. (2017). Is body dissatisfaction changing across time? A cross-temporal meta-analysis. Psychol. Bull..

[32] Wertheim E.H., Paxton S.J., Cash T.F., Smolak L. (2011). Body Image Development in Adolescent Girls. Body Image. A Handbook of Science, Practice, and Prevention.

[33] Ricciardelli L.A., McCabe M.P., Cash T.F., Smolak L. (2011). Body Image Development in Adolescent boys. Body Image. A Handbook of Science, Practice, and Prevention.

[34] Lantz E.L., Gaspar M.E., DiTore R., Piers A.D., Schaumberg K. (2018). Conceptualizing body dissatisfaction in eating disorders within a self-discrepancy framework: A review of evidence. Eat. Weight Disord..

[35] Bucchianeri M.M., Arikian A.J., Hannan P.J., Eisenberg M.E., Neumark-Sztainer D. (2013). Body dissatisfaction from adolescence to young adulthood: Findings from a 10-year longitudinal study. Body Image.

[36] Jankauskiene R., Baceviciene M. (2019). Body image concerns and body weight overestimation do not promote healthy behaviour: Evidence from adolescents in Lithuania. Int. J. Environ. Res. Public Health.

[37] Coelho E.M., Fonseca S.C., Pinto G.S., Mourão-Carvalhal M.I. (2016). Factors associated with body image dissatisfaction in Portuguese adolescents: Obesity, sports activity and TV watching. Motricidade.

[38] Kantanista A., Król-Zielińska M., Borowiec J., Osiński W. (2017). Is underweight associated with more positive body image? Results of a cross-sectional study in adolescent girls and boys. Span. J. Psychol..

[39] Fernández-Bustos J.G., Infantes-Paniagua Á., Gonzalez-Martí I., Contreras-Jordán O.R. (2019). Body Dissatisfaction in Adolescents: Differences by Sex, BMI and Type and Organisation of Physical Activity. Int. J. Environ. Res. Public Health.

[40] Wang Y., Lynne S.D., Witherspoon D., Black M.M. (2020). Longitudinal bidirectional relations between body dissatisfaction and depressive symptoms among Black adolescents: A cross-lagged panel analysis. PLoS ONE.

[41] Higgins E.T. (1987). Self-discrepancy: A theory relating self and affect. Psychol. Rev..

[42] Stevens E.N., Lovejoy M.C., Pittman L.D. (2014). Understanding the relationship between actual: Ideal discrepancies and depressive symptoms: A developmental examination. J. Adolesc..

[43] Strauman T.J. (2017). Self-regulation and psychopathology: Toward an integrative translational research paradigm. Annu. Rev. Clin. Psychol..

[44] Solomon-Krakus S., Sabiston C.M., Brunet J., Castonguay A.L., Maximova K., Henderson M. (2017). Body image self-discrepancy and depressive symptoms among early adolescents. J. Adolesc. Health.

[45] Cortese S., Falissard B., Angriman M., Pigaiani Y., Banzato C., Bogoni G., Pellegrino M., Cook S., Pajno-Ferrara F., Bernardina B.D. (2009). The relationship between body size and depression symptoms in adolescents. J. Pediatr..

[46] Quek Y.H., Tam W.W., Zhang M.W., Ho R.C. (2017). Exploring the association between childhood and adolescent obesity and depression: A meta-analysis. Obes. Rev..

[47] Richard A., Rohrmann S., Lohse T., Eichholzer M. (2016). Is body weight dissatisfaction a predictor of depression independent of body mass index, sex and age? Results of a cross-sectional study. BMC Public Health.

[48] Chen G., Guo G., Gong J., Xiao S. (2015). The association between body dissatisfaction and depression: An examination of the moderating effects of gender, age, and weight status in a sample of Chinese adolescents. J. Psychol. Couns. Sch..

[49] Inchley J., Currie D., Budisavljevic S., Torsheim T., Jåstad A., Cosma A., Kelly C., Arnarsson A.M., Samdal O. (2020). Spotlight on Adolescent Health and Well-Being. Findings from the 2017/2018 Health Behaviour in School-Aged Children (HBSC) Survey in Europe and Canada.

[50] Leal I.P., Antunes R., Passos T., Pais-Ribeiro J., Maroco J. (2009). Study of the Depression, Anxiety and Stress Scales for children (DASS). Psicol. Saúde Doenças.

[51] Lovibond P., Lovibond S. (1995). The structure of negative emotional states: Comparison of the depression anxiety stress scales (DASS) with the Beck Depression and Anxiety Inventories. Behav. Res. Ther..

[52] Francisco R., Narciso I., Alarcão M. (2012). Satisfaction with body image in Portuguese adolescents and adults: Contributions to the Contour Drawing Rating Scale validation process. Rev. Iberoam. Diagn. Eval. Psicol..

[53] Thompson M.A., Gray J.J. (1995). Development and validation of a new body-image assessment scale. J. Personal. Assess..

[54] Bulik C.M., Wade T.D., Heath A.C., Martin N.G., Stunkard A.J., Eaves L.J. (2001). Relating body mass index to gural stimuli: Population-based normative data for Caucasians. Int. J. Obes. Relat. Metab. Disord..

[55] Goodman E., Hinden B.R., Khandelwal S. (2000). Accuracy of teen and parental reports of obesity and body mass index. Pediatrics.

[56] Fonseca H., Silva A.M., Matos M.G., Esteves I., Costa P., Guerra A., Gomes-Pedro J. (2010). Validity of BMI based on self-reported weight and height in adolescents. Acta Paediatr..

[57] de Onis M., Onyango A.W., Borghi E., Siyam A., Nishida C., Siekmann J. (2007). Development of a WHO growth reference for school-aged children and adolescents. Bull. WHO.

[58] Blossner M., Siyam A., Borghi E., Onyango A., de Onis M. (2009). WHO AnthroPlus for Personal Computers Manual: Software for Assessing Growth of the World’s Children and Adolescents.

[59] Cuesta M., Fonseca-Pedrero E., Vallejo G., Muñiz J. (2013). Missing data and psychometric properties in personality tests. An. Psicol..

[60] Hayes A.F. (2018). Introduction to Mediation, Moderation, and Conditional Process Analysis: A Regression-Based Approach.

[61] Berry W.D. (1993). Understanding Regression Assumptions.

[62] Cohen J. (1988). Statistical Power Analysis for the Behavioral Sciences.

[63] Carvalho C., Cunha M., Cherpe S., Galhardo A., Couto M. (2015). Validation of the Portuguese version of the Center for Epidemiologic Studies Depression Scale for Children (CES-DC). Rev. Port. Investig. Comport. Soc..

[64] Kwong A.S., Manley D., Timpson N.J., Pearson R.M., Heron J., Sallis H., Stergiakouli E., Davis O.S.P., Leckie G. (2019). Identifying critical points of trajectories of depressive symptoms from childhood to young adulthood. J. Youth Adolesc..

[65] Monteiro S., Matos A.P., Oliveira S. (2015). The moderating effect of gender: Traumatic experiences and depression in adolescence. Procedia Soc. Behav. Sci..

[66] Wade T.J., Cairney J., Pevalin D.J. (2002). Emergence of gender differences in depression during adolescence: National panel results from three countries. J. Am. Acad. Child Adolesc. Psychiatry.

[67] Hankin B.L., Abramson L.Y., Moffitt T.E., Silva P.A., McGee R., Angell K.E. (1998). Development of depression from preadolescence to young adulthood: Emerging gender differences in a 10-year longitudinal study. J. Abnorm. Psychol..

[68] Patrick J., Dyck M., Bramston P. (2010). Depression Anxiety Stress Scale: Is it valid for children and adolescents?. J. Clin. Psychol..

[69] Bennett D.S., Ambrosini P.J., Kudes D., Metz C., Rabinovich H. (2005). Gender differences in adolescent depression: Do symptoms differ for boys and girls?. J. Affect. Disord..

[70] Bulhões C., Ramos E., Severo M., Dias S., Barros H. (2019). Measuring depressive symptoms during adolescence: What is the role of gender?. Epidemiol. Psychiatr. Sci..

[71] Boutelle K., Neumark-Sztainer D., Story M., Resnick M. (2002). Weight control behaviors among obese, overweight, and nonoverweight adolescents. J. Pediatr. Psychol..

[72] Pinquart M., Silbereisen R.K., Wiesner M. (2004). Changes in discrepancies between desired and present states of developmental tasks in adolescence: A 4-process model. J. Youth Adolesc..

[73] Harter S. (2012). The Construction of the Self: Developmental and Sociocultural Foundations.

[74] Revah-Levy A., Speranza M., Barry C., Hassler C., Gasquet I., Moro M.R., Falissard B. (2011). Association between Body Mass Index and depression: The “fat and jolly” hypothesis for adolescents girls. BMC Public Health.

[75] Chang L.Y., Chang H.Y., Wu W.C., Lin L.N., Wu C.C., Yen L.L. (2017). Body mass index and depressive symptoms in adolescents in Taiwan: Testing mediation effects of peer victimization and sleep problems. Int. J. Obes..

[76] Oddy W.H., Allen K.L., Trapp G.S., Ambrosini G.L., Black L.J., Huang R.C., Rzehak P., Runions K.C., Pan F., Beilin L.J. (2018). Dietary patterns, body mass index and inflammation: Pathways to depression and mental health problems in adolescents. Brain Behav. Immun..

[77] Hoare E., Skouteris H., Fuller-Tyszkiewicz M., Millar L., Allender S. (2014). Associations between obesogenic risk factors and depression among adolescents: A systematic review. Obes. Rev..

[78] Hankin B.L. (2017). Depression during childhood and adolescence. The Oxford Handbook of Mood Disorders.

[79] McCabe M.P., Ricciardelli L.A. (2004). Body image dissatisfaction among males across the lifespan: A review of past literature. J. Psychosom. Res..

[80] Olfert M.D., Barr M.L., Charlier C.M., Famodu O.A., Zhou W., Mathews A.E., Byrd-Bredbenner C., Colby S.E. (2018). Self-reported vs. measured height, weight, and BMI in young adults. Int. J. Environ. Res. Public Health.

[81] Hudson G.M., Lu Y., Zhang X., Hahn J., Zabal J.E., Latif F., Philbeck J. (2020). The development of a BMI-guided shape morphing technique and the effects of an individualized figure rating scale on self-perception of body size. Eur. J. Investig. Health Psychol. Educ..

[82] Austin S.B., Haines J., Veugelers P.J. (2009). Body satisfaction and body weight: Gender differences and sociodemographic determinants. BMC Public Health.

[83] Heinicke B.E., Paxton S.J., McLean S.A., Wertheim E.H. (2007). Internet-delivered targeted group intervention for body dissatisfaction and disordered eating in adolescent girls: A randomized controlled trial. J. Abnorm. Child Psychol..

